# Exosome Analysis in Tumor-Draining Pulmonary Vein Identifies NSCLC Patients with Higher Risk of Relapse after Curative Surgery

**DOI:** 10.3390/cancers11020249

**Published:** 2019-02-21

**Authors:** Alfons Navarro, Laureano Molins, Ramon M. Marrades, Jorge Moises, Nuria Viñolas, Sara Morales, Jordi Canals, Joan J. Castellano, José Ramírez, Mariano Monzo

**Affiliations:** 1 Molecular Oncology and Embryology Laboratory, Human Anatomy Unit, School of Medicine, University of Barcelona, IDIBAPS, 08036 Barcelona, Spain; saramolo.19@gmail.com (S.M.); canals.serrat@ub.edu (J.C.); joan.castellano@ub.edu (J.J.C.); 2 Department of Thoracic Surgery, Institut Clínic de Respiratori (ICR), Hospital Clínic de Barcelona, University of Barcelona, 08036 Barcelona, Spain; lmolins@clinic.cat; 3 Department of Pneumology, Institut Clínic de Respiratori (ICR), Hospital Clínic de Barcelona, University of Barcelona, IDIBAPS, CIBER de Enfermedades Respiratorias (CIBERES), 08036 Barcelona, Spain; marrades@clinic.cat (R.M.M.); jrmoises@clinic.cat (J.M.); 4 Department of Medical Oncology, Institut Clínic Malalties Hemato-Oncològiques (ICMHO), Hospital Clínic de Barcelona, University of Barcelona, IDIBAPS, 08036 Barcelona, Spain; nvinolas@clinic.cat; 5 Department of Pathology, Centro de Diagnóstico Biomédico (CDB), Hospital Clínic de Barcelona, University of Barcelona, IDIBAPS, CIBERES, 08036 Barcelona, Spain; jramirez@clinic.cat

**Keywords:** exosome, extracellular vesicles, tumor-draining vein, relapse, lung cancer, NSCLC

## Abstract

Since tumor-draining pulmonary vein blood (PV) is enriched in tumor-secreted products, we hypothesized that it would also be enriched in tumor-derived exosomes, which would be important in the metastasis process. We characterized exosomes from PV of 61 resected non-small cell lung cancer (NSCLC) patients to evaluate its potential as relapse biomarkers. Exosomes were characterized using transmission electron microscopy, western blot and nanoparticle tracking analysis and we examined time to relapse (TTR) and overall survival (OS). Differences between PV and peripheral vein were found. PV was enriched in smaller exosomes than the paired peripheral vein (*p* = 0.01). Moreover, PV exosome size mode was able to identify relapsed patients (Area under the curve [AUC] = 0.781; 95%CI: 0.6641–0.8978), in whom exosome size was smaller (<112 nm; *p* < 0.001). The combination of PV exosome size and N (lymph node involvement) showed an AUC of 0.89 (95%CI: 0.80–0.97). Moreover, smaller PV exosome size was associated with shorter TTR (28.3 months vs. not reached, *p* < 0.001) and OS (43.9 months vs. not reached, *p* = 0.009). Multivariate analyses identified PV exosome size and stage as independent prognostic markers for TTR and OS. PV exosome size is a promising relapse biomarker after surgery that can add valuable information to clinical variables.

## 1. Introduction

Treatment of non-small cell lung cancer (NSCLC) depends on disease stage. In early and locally advanced stages, surgical resection of the tumor is, when possible, the first option and is considered curative [1]. In stage II and IIIa, adjuvant chemotherapy after surgery has been shown to improve survival [2]. However, the prognosis of these patients is still dismal—five-year survival rates of 60–80% for stage I, 20–50% for stage II and 23% for stage IIIA patients—due to high relapse rates (40%) after surgery. One of the factors associated with patient relapse is the presence of circulating tumor cells (CTCs) in peripheral blood, which has been associated with worse prognosis [3]. However, studies in peripheral blood are limited by the low number of CTCs that can be detected, leading some investigators to analyze the utility of detecting CTCs and other biomarkers in blood from the tumor-draining pulmonary vein, which can better identify patients with a high risk of relapse after surgery [4]. While these results are interesting, the metastatic process cannot be explained only by the presence of CTCs, since their survival in the bloodstream, the extravasation process and the anchorage to the metastatic site are challenging. Recently, it has been shown that extracellular vesicles signals emitted by the primary tumor that are picked up by specific cells with the appropriate receptors [5], can contribute to the oncogenesis and metastasis process. The target cells can be located either in the local microenvironment of the tumor or in distant organs, since exosomes can reach the bloodstream. Extracellular vesicles are involved in several biological functions, including cross-talk between cells, tumorogenesis, drug resistance, angiogenesis promotion, and metastasis [6,7]. The extracellular vesicles are classified according to size and biogenesis as exosomes (<150 nm and produced from multivesicular bodies) or microvesicles (>150 nm and formed through the direct budding of the plasma membrane) [7]. Exosomes have been shown to play a role in the metastatic process through the creation of a pre-metastatic niche that facilitates the anchorage and growth of CTCs in the metastatic site [8]. Recently, it has been also shown in vitro and in vivo in liver cancer that primary-tumor derived exosomes can deliver their cargo on CTCs and enhance their adhesion supporting metastasis formation [9]. 

To our knowledge, most of the studies have been focused on the study of exosome cargo to identify diagnosis/prognosis biomarkers, such as exosomal microRNAs [10] or exosomal proteins [11], and few studies have analyzed whether exosome levels themselves could be used as a disease parameter. Recently, König et al. have shown that the analysis of extracellular vesicles concentration in blood of breast cancer patients may serve as a complementary parameter reflecting the status of minimal residual disease as well as therapy and disease outcome in parallel with CTC investigation [12]. 

We hypothesized that the quantification and size distribution of exosomes in circulation could be an indicator of tumor metastatic capacity in non-small cell lung cancer. However, we speculate that tumor-derived exosomes in peripheral blood will be very diluted in comparison with tumor-draining pulmonary vein as previously observed with CTCs, especially in early-stage patients [13,14,15]. Therefore, we hypothesized that the analysis of tumor-derived exosomes could provide more reliable results in blood obtained from the pulmonary vein, where the products released by the tumor would be concentrated. We have compared the quantity and size of exosomes from tumor-draining pulmonary and paired peripheral vein and evaluated their potential as a biomarker of relapse in NSCLC.

## 2. Results

### 2.1. Patients

The analysis included 61 patients (Table 1). Twenty-three patients (37.7%) received adjuvant cisplatin-based chemotherapy (2 stage IB, 16 stage II, and 5 stage III). None of the patients received neoadjuvant treatment. Median follow-up time was 40.87 months (IQR: 23.53–49.27). Twenty-one patients (34.4%) relapsed after surgery, 15 with distant metastases. Seventeen patients (27.8%) died during follow-up; although the cause of death was not recorded, 14 (82.4%) of them died after relapse while three (17.6%) died without relapse, suggesting that lung cancer was the main cause of death (*p* < 0.001). Pulmonary vein blood was obtained from the entire cohort, but blood from the peripheral vein was only obtained from 55 of the 61 patients (92%).

### 2.2. Exosome Characterization

**Figure 1:**
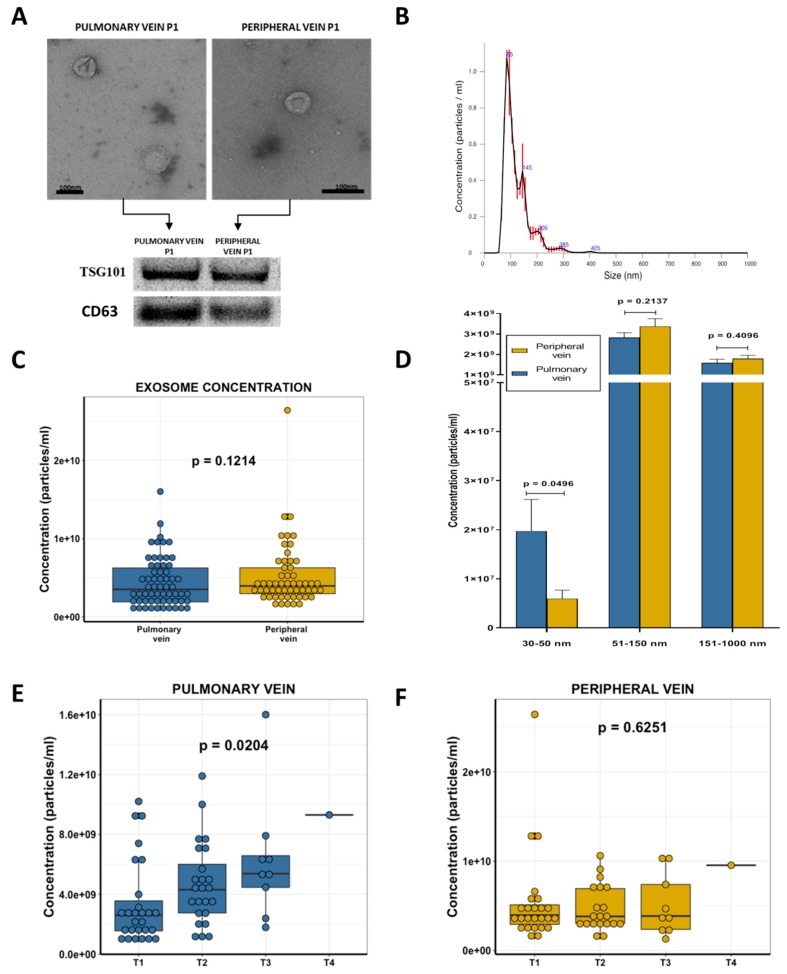
(**A**) Exosomes were characterized by transmission electron microscopy using negative staining and Western blot using the exosome marker TSG101. (**B**) The quantification and size analysis were performed by Nanosight. An example of the size distribution graph obtained is shown. (**C**) Boxplot showing exosome levels in pulmonary and peripheral vein in non-small cell lung cancer (NSCLC) patients. (**D**) Bar plot showing exosomal size levels in pulmonary and peripheral vein according to three size groups: 30–50 nm, 51–150 nm and 151–1000 nm. (**E**) Boxplot showing pulmonary vein exosome levels according to T stage in NSCLC patients. (**F**) Boxplot showing peripheral vein exosome levels according to T stage in NSCLC patients.

Transmission electron microscopy (TEM) morphological analysis showed the presence of round 27–200 nm vesicles. To further validate that these vesicles were, at least in part, exosomes, the presence of the established exosomal markers TSG101 and CD63 was determined using western blot, which confirmed the presence of the exosomes (Figure 1A). Using nanoparticle tracking analysis (NTA), which uses the properties of both Brownian motion and light scattering to obtain the particle size distribution of samples in a liquid suspension (Video S1), we obtained a size distribution profile graph (Figure 1B), and concentration (nanoparticles/mL) and size measures for each sample. The median concentration in all samples by NTA was 3.815 × 10^9^ particles per mL (range: 7.72 × 10^8^–2.64 × 10^10^) and the mode of particle diameter was 114.5 nm (range: 85.1–173.3). 

### 2.3. Exosome Concentration and Clinical Characteristics

When we compared the overall exosome concentration between the pulmonary and peripheral veins, no significant differences were observed (*p* = 0.1214, Figure 1C). However, the sub-analysis of the specific levels of different exosome sizes showed that the pulmonary vein was enriched in exosomes of 30–50 nm (*p* = 0.0496) while no significant differences were observed for other group sizes (51–150/151–1000 nm, Figure 1D).

The correlation of the overall exosome concentration with the main clinical characteristics of the patients showed that the quantity of exosomes in the pulmonary vein was related to the T stage (of the TNM classification), where T1 patients had significantly fewer exosomes than others (*p* = 0.0204, Figure 1E). However, this association was not observed in the paired blood from the peripheral vein (*p* = 0.6251, Figure 1F). Moreover, when we grouped patients according to median tumor size, those with tumors smaller than 35 mm had fewer pulmonary vein exosomes than those with larger tumors (*p* = 0.01). No significant association was observed in the peripheral vein.

### 2.4. Pulmonary Vein Exosome Size Identifies Patients Who Relapse after Curative Surgery 

**Figure 2:**
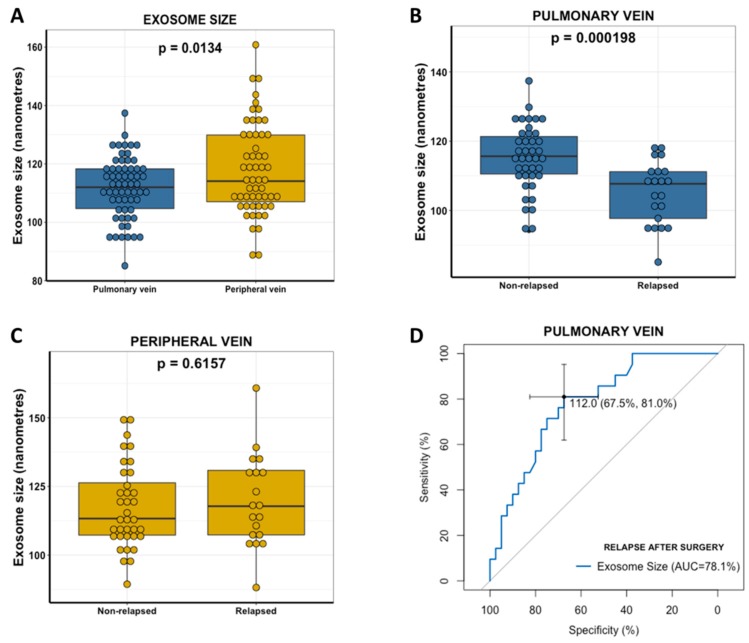
The relationship between exosomal size and relapse after surgery in NSCLC patients. (**A**) Boxplot showing exosome size distribution (size mode) in pulmonary and peripheral vein in NSCLC patients. (**B**) Boxplot showing pulmonary vein exosomal size in relapsed and non-relapsed NSCLC patients. (**C**) Boxplot showing peripheral vein exosomal size in relapsed and non-relapsed NSCLC patients. (**D**) ROC curve analysis of pulmonary vein exosomal size values predicting relapse after surgery in NSCLC patients. The cut-off for disease relapse after surgery was set at 112 nm. The area under the curve of 0.78 with pulmonary vein exosomal size values of <112 nm resulted in a sensitivity of 81% and specificity of 67.5%.

Significant differences in exosome size between pulmonary and peripheral veins were observed (Figure 2A). Pulmonary vein exosomes were significantly smaller than those from the paired peripheral vein (mean mode size 111.8754 vs. 118.1545; *p* = 0.0134). 

The size of exosomes from the pulmonary vein was significantly smaller in relapsed patients than in non-relapsed patients (mean size 105.26 vs. 115.35 nm, *p* < 0.001) (Figure 2B). Nevertheless, these differences in size were not observed in the peripheral vein (*p* = 0.62, Figure 2C).

ROC curves were generated to investigate exosome size as a predictive biomarker of relapse after surgery. The area under the curve (AUC) value according to pulmonary vein exosome size was 0.78 (95% confidence interval (CI), 0.6641–0.8978) with a sensitivity of 67.5% and specificity of 81% in distinguishing patients who relapse after surgery in its best threshold (112 nm) (Figure 2D). Using 112 nm as a threshold, the negative predictive value was 87.1%, and the positive predictive value was 56.7%.

In order to compare the impact of the pulmonary vein exosome size with other clinical factors, lymph node involvement (N), and disease stage were included in the analysis. N showed an AUC of 0.76 (95% CI: 0.64–0.88), and disease stage showed an AUC of 0.71 (95% CI, 0.59–0.84). To verify that no other confounding parameters could have affected these results, we performed a binary logistic regression multivariate analysis for relapse to verify the independent impact of pulmonary vein exosome size to predict relapse after curative surgery. Pulmonary vein exosome size (hazard ratio (HR), 0.87 (95%CI, 0.79–0.94); *p* = 0.0019) and N (HR, 7.1 (95% CI, 2.48–27.14); *p* = 0.0011) emerged as independent markers of relapse. Interestingly, when we combined pulmonary vein exosome size and N, a good AUC of 0.89 (95% CI, 0.80–0.97) was obtained. 

### 2.5. Pulmonary Vein Exosome Size Is Associated with Outcome after Curative Surgery 

**Figure 3:**
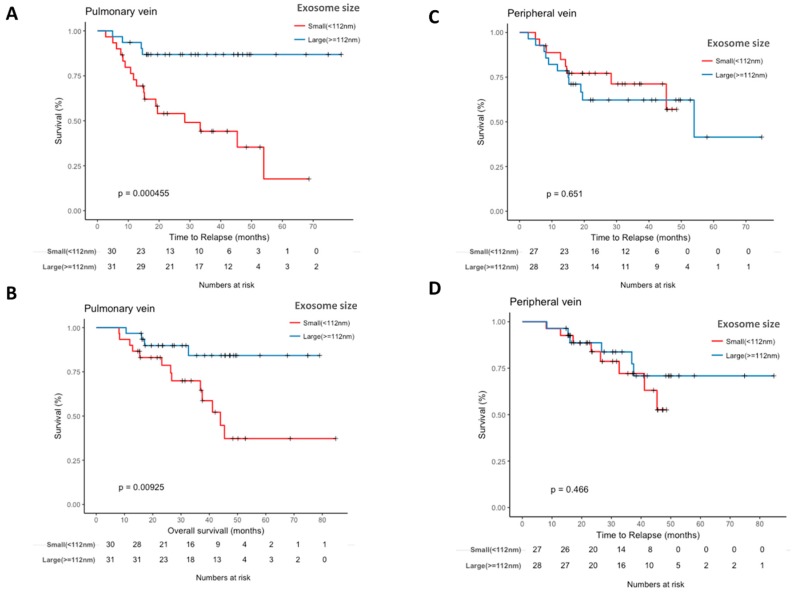
Kaplan-Meier survival analysis of associations between exosomal size and time to relapse (TTR) and overall survival (OS) among patients with NSCLC. (**A**) TTR according to pulmonary vein exosomal size. (**B**) OS according to pulmonary vein exosomal size. (**C**) TTR according to peripheral vein exosomal size. (**D**) OS according to peripheral vein exosomal size.

The smaller exosome size (<112 nm) in the pulmonary vein was associated with shorter TTR (28.3 months vs. not reached; *p* = 0.0004; Figure 3A) and shorter OS (43.9 months vs. not reached; *p* = 0.0092; Figure 3B). No significant differences were observed when exosome size was analyzed in the peripheral vein (Figure 3C,D). 

**Figure 4:**
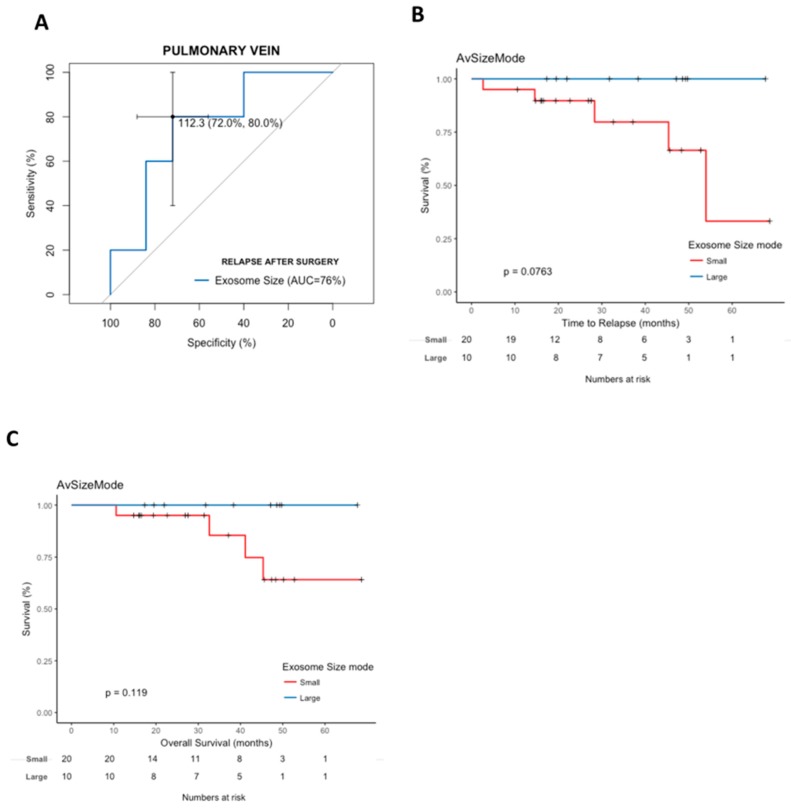
(**A**) ROC curve analysis of pulmonary vein exosomal size values predicting relapse after surgery in stage I NSCLC patients. The cut-off for relapse was set at 112.3 nm. The area under the curve of 0.76 with pulmonary vein exosomal size values of <112.3 nm resulted in a sensitivity of 80% and specificity of 72%. (**B**) Kaplan-Meier survival analysis of associations between pulmonary vein exosomal size and time to relapse (TTR) among patients with stage I NSCLC. (**C**) Kaplan-Meier survival analysis of the association between pulmonary vein exosomal size and overall survival (OS) among patients with stage I NSCLC.

An exploratory analysis of patients with stage I disease (*n* = 30) showed that exosome size discriminated stage I patients with a high relapse risk (AUC=0.76 (95% CI, 0.53–0.98)) (Figure 4A). In addition, a trend towards shorter TTR (*p* = 0.0763) and OS (*p* = 0.1190) was observed for patients with smaller exosome size (Figure 4B,C).

### 2.6. Cox Modeling of Relapse and Survival

In the multivariate Cox analyses, exosome size emerged as an independent risk factor for TTR (HR, 6.66 (95%CI, 2.06–21.51); *p* = 0.0015) and OS (HR, 4.55 (95%CI, 1.43–14.49); *p* = 0.0104). Stage was also an independent marker of TTR (HR, 2.42 (95% CI, 1.16–5.03); *p* = 0.0180) and OS (HR, 2.93 (95% CI, 1.46–5.84); *p* = 0.0024) (Table 2).

## 3. Discussion

In the present study, we have examined exosome size distribution and levels to evaluate their potential to predict relapse of resected NSCLC patients. Importantly, exosomes were analyzed in blood obtained from the pulmonary vein during surgery and compared with those obtained from peripheral blood before tumor resection. Previous studies showed that blood obtained from a tumor-draining vein is enriched in tumor-secreted products, including CTCs [13], which led us to hypothesize that it would be enriched in primary tumor-derived exosomes. Our results showed that pulmonary vein blood was enriched in exosomes of 30–50 nm, while no differences were observed in other size ranges. Remarkably, pulmonary vein overall exosome levels were more highly correlated with tumor characteristics than those in peripheral blood. In this line, we observed a significant association between T stage and pulmonary vein—but not peripheral vein—exosome levels. T stage is related to tumor size and we observed higher pulmonary vein exosome levels in tumors larger than the median (35 mm). Pulmonary vein exosome analysis may more closely represent tumor characteristics than the analysis of peripheral vein. 

Moreover, significant differences in exosome size distribution (mode of size) were observed between the pulmonary and peripheral veins, where pulmonary vein exosomes were smaller than peripheral vein exosomes. Interestingly, the size of pulmonary vein exosomes correlated with the risk of post-surgical relapse and survival. Patients with smaller pulmonary vein exosomes had a higher risk of relapse and shorter TTR and OS, while no differences were observed according to peripheral vein exosome size. In line with our results, an in vitro study in prostate cancer found that the amount and size of exosomes changed after the acquisition of resistance to docetaxel treatment, where the size of exosomes secreted by resistant cells was smaller than of those secreted by the sensitive cells [5]. Recently, Kim et al. reported that extracellular vesicle size was an independent predictor of progression-free survival at first response assessment to chemotherapy in pancreatic cancer [16]. Taken together with our results, these findings allow us to speculate that a more aggressive cell phenotype is linked to the production of smaller exosomes.

The evaluation of pulmonary vein exosome size was able to identify patients who relapsed with an accuracy of 72%. The AUC of 0.78 for exosome size observed in our cohort is fair and was able to discriminate relapsed patients more accurately than disease stage (AUC of 0.71), or N (AUC of 0.76). Interestingly, the good AUC of 0.89 for the combination of exosome size and N suggest that exosome size may potentially complement clinical variables in identifying patients with a high risk of relapse, who could benefit from more aggressive treatment. This is especially relevant for stage I patients, who do not receive any post-surgical treatment, in comparison with stage II–IIIa patients who benefit from platinum-based chemotherapy [1]. In a sub-analysis of stage I patients, exosome size as a continuous variable also discriminated patients with a high relapse risk and shorter TTR (Cox univariate HR, 0.91 (95% CI, 0.829–0.9998); *p* = 0.0496). These preliminary results, if confirmed in larger studies, indicate that pulmonary vein exosome size is a promising biomarker of relapse for stage I patients. 

Despite the benefits described in our work and others [13,14,15] of the analysis of tumor-secreted products in tumor-draining pulmonary vein, the obtaining of this type of sample could be considered invasive in comparison with peripheral blood analysis. However, we have to take into account that this analysis is limited to surgical NSCLC patients, where the access to the pulmonary vein is neither modifying the routine surgical procedure nor requiring additional lung dissection. The tumor-draining pulmonary vein is punctured immediately on opening the chest prior to beginning the resection of the tumor. This approach was safe with no complications reported in our cohort of patients or in previous studies [13]. Moreover, most of the resected NSCLC patients are early-stage (I–II) patients in which the number of tumor-secreted products in peripheral blood is more limited, and, therefore, they will be the most benefited from the tumor-draining pulmonary vein analysis.

Several authors have found that exosomes and their cargo, analyzed in peripheral blood [11,17,18] and bronchoalveolar lavage [19], can be useful diagnostic biomarkers in NSCLC. However, to our knowledge, this is the first report to analyze exosomes in blood from the pulmonary vein and to show that they can be used as an independent prognostic biomarker for relapse and survival after surgery in NSCLC. Further investigation in a larger cohort of patients is warranted to validate these findings and explore the potential for exosome size analysis to be used in the clinical management of resected NSCLC patients.

## 4. Material and Methods

### 4.1. Patient Samples

The study included 61 stage I-IIIa NSCLC patients who underwent complete surgical resection in our institution (Table 1). Blood (4 mL in EDTA tube) from the tumor-draining pulmonary vein was obtained during the surgery before tumor resection or vessel ligation. Blood from a peripheral vein was obtained by the nurse anesthesiologist before the start of surgery. Plasma obtained by centrifugation was stored at −80 °C until processing. Written informed consent was obtained from each participant in accordance with the Declaration of Helsinki and the study was approved by the Clinical Research Ethics Committee of the Hospital Clínic de Barcelona (project approval number HCB/2017/1052). 

### 4.2. Exosome Purification and Characterization

Exosomes were isolated from 200 μL of plasma by ultracentrifugation in a Sorvall MX Plus Micro-Ultracentrifuge with S140AT Rotor as previously described [20]. Pelleted exosomes resuspended in 200 μL of dPBS: 100 μL were used for exosome characterization by TEM and Western blot and 100 μL were used for exosome quantification and size analysis by NTA. Exosomal marker TSG101 was analyzed by Western blot as previously described [20,21], and CD63 was analyzed using Rabbit polyclonal to CD63 (ab68418, from Abcam, Cambridge, UK). For TEM analysis, exosomes in dPBS were fixed in a final concentration of 2% paraformaldehyde, mounted on copper-mesh formvar grids and negatively stained by 2% uranyl acetate. Samples were observed using a TEM JEOL J1010 80 kV at the Electron Cryomicroscopy Unit of the University of Barcelona (CCiTUB, Barcelona, Spain). NTA analysis was performed on a Nanosight NS300 in the ICTS “NANBIOSIS” (Biomaterial Processing and Nanostructuring Unit of the CIBER in Bioengineering, Biomaterials & Nanomedicine at Institut de Ciència de Materials de Barcelona, CSIC, Campus UAB, Bellaterra, Barcelona, Spain).

### 4.3. Statistical Analysis

Assumptions of distributional normality were tested using the Shapiro-Wilk test and quantile-quantile plot. Continuous data were tested with the T-test (two groups) or ANOVA (more than two groups) when normally distributed and the Mann–Whitney U test or Kruskal Wallis test when not normally distributed. Receiver operating characteristic (ROC) curves were calculated using R package pROC. Time to relapse (TTR) was calculated as the time between resection and recurrence or last follow-up and overall survival (OS) as the time between resection and death from any cause or last follow-up. Kaplan-Meier curves for TTR and OS were drawn and compared by means of a log-rank test. All clinical factors with *p* < 0.1 in the univariate analysis were included in the Cox multivariate regression analyses. Statistical significance was set at *p* ≤ 0 .05. All statistical analyses were performed using R v3.3 (*R* Foundation for Statistical Computing, https://www.r-project.org/). 

## 5. Conclusions

The evaluation of tumor-draining pulmonary vein exosome size is a promising prognostic biomarker for relapse and survival after surgery and can add valuable information to clinical variables. 

## Figures and Tables

**Table 1:** Main clinical characteristics of the patients and univariate *p*-values (log-rank test) for time to relapse (TTR) and overall survival (OS).

Characteristics	Value	*N* = 61	TTR	OS
		*N* (%)	*p*-Value	*p*-Value
Sex	Male	41 (67.2)		
	Female	20 (32.8)	0.5427	0.6291
Age, y	Mean (Range)	63 (32–80)		
	≤65	34 (55.7)		
	>65	27 (44.3)	0.8	0.2253
ECOG PS	0	26 (42.6)		
	1	35 (57.4)	0.4723	0.3479
Pathological Stage	I	30 (49.2)		
	II	24 (39.3)		
	III	7 (11.5)	**0.0069**	**0.0082**
Histology	Adenocarcinoma	35 (57.4)		
	Squamous cell carcinoma	16 (26.2)		
	Others	10 (16.4)	0.7562	0.9579
Type of surgery	Segmentectomy	5 (8.2)		
	Lobectomy/bilobectomy	48 (78.7)		
	Pneumonectomy	7 (11.5)		
	Atypical Resection	1 (1.6)	0.3963	0.4178
Smoking history	Current Smoker	32 (52.5)		
	Former Smoker	24 (39.3)		
	Never smoker	5 (8.2)	0.1118	0.1881
FEV_1_	Liters (±SD)	2.4 (±0.6)		
	%pred (±SD)	76.8 (±16.1)	-	-
FVC	Liters (±SD)	3.56 (±0.74)		
	%pred (±SD)	88 (±16.42)	-	-
FEV_1_/FVC	Ratio (±SD)	67.2 (±9.74)	-	-
Tumor size	mm (±SD)	38.3 (±23.2)	-	-
Received adjuvant chemotherapy	Yes	23 (37.7)		
	No	38 (62.3)	**0.0113**	0.1958
Experienced recurrence	No	40 (65.6)		
	Yes	21 (34.4)	-	-

ECOG PS, Eastern Cooperative Oncology Group performance status; FEV_1_, forced expiratory volume in 1 s; FVC, forced vital capacity. Bold for *p* < 0.05.

**Table 2:** Cox multivariate analyses of time to relapse and overall survival. Bold for *p* < 0.05.

Time to Relapse	Hazard Ratio (HR, 5% CI)	*p*-Value
Stage	2.42 (1.16–5.03)	**0.0180**
Adjuvant chemotherapy	1.59 (0.59–4.26)	0.3563
Exosome size in pulmonary vein <112 nm	6.66 (2.06–21.51)	**0.0015**
**Overall Survival**	**HR (95% CI)**	***p*-value**
Stage	2.93 (1.46–5.84)	**0.0024**
Exosome size in pulmonary vein <112 nm	4.55 (1.43–14.49)	**0.0104**

## Supplementary Materials

The following are available online at https://www.mdpi.com/2072-6694/11/2/249/s1, Video S1. Video captured during the nanoparticle tracking analysis (NTA) measurement in Nanosight NS300 equipment showing the movement of particles (exosomes) in suspension.
